# An Analysis of the Cross-Reactivity of Autoantibodies to GAD65 and GAD67 in Diabetes

**DOI:** 10.1371/journal.pone.0018411

**Published:** 2011-04-08

**Authors:** Bindu Jayakrishnan, David E. Hoke, Christopher G. Langendorf, Ashley M. Buckle, Merrill J. Rowley

**Affiliations:** Department of Biochemistry and Molecular Biology, Monash University, Clayton, Victoria, Australia; Universita di Sassari, Italy

## Abstract

**Background:**

Autoantibodies to GAD65 (anti-GAD65) are present in the sera of 70–80% of patients with type 1 diabetes (T1D), but antibodies to the structurally similar 67 kDa isoform GAD67 are rare. Antibodies to GAD67 may represent a cross-reactive population of anti-GAD65, but this has not been formally tested.

**Methodology/Principal Findings:**

In this study we examined the frequency, levels and affinity of anti-GAD67 in diabetes sera that contained anti-GAD65, and compared the specificity of GAD65 and GAD67 reactivity. Anti-GAD65 and anti-GAD67 were measured by radioimmunoprecipitation (RIP) using ^125^I labeled recombinant GAD65 and GAD67. For each antibody population, the specificity of the binding was measured by incubation with 100-fold excess of unlabeled GAD in homologous and heterologous inhibition assays, and the affinity of binding with GAD65 and GAD67 was measured in selected sera. Sera were also tested for reactivity to GAD65 and GAD67 by immunoblotting. Of the 85 sera that contained antibodies to GAD65, 28 contained anti–GAD67 measured by RIP. Inhibition with unlabeled GAD65 substantially or completely reduced antibody reactivity with both ^125^I GAD65 and with ^125^I GAD67. In contrast, unlabeled GAD67 reduced autoantibody reactivity with ^125^I GAD67 but not with ^125^I GAD65. Both populations of antibodies were of high affinity (>10^10^ l/mol).

**Conclusions:**

Our findings show that autoantibodies to GAD67 represent a minor population of anti-GAD65 that are reactive with a cross-reactive epitope found also on GAD67. Experimental results confirm that GAD65 is the major autoantigen in T1D, and that GAD67 *per se* has very low immunogenicity. We discuss our findings in light of the known similarities between the structures of the GAD isoforms, in particular the location of a minor cross-reactive epitope that could be induced by epitope spreading.

## Introduction

Glutamic acid decarboxylase 65 (GAD65), a neuroendocrine enzyme, is a key autoantigen in type 1 diabetes (T1D) [1], in Latent Autoimmune Diabetes of Adults (LADA) [2] and in various neurological diseases [3], [4], [5], [6], [7]. Serum autoantibodies to GAD65 are an important marker in the early prediction and diagnosis of T1D [8], [9]. The closely related 67 kDa isoform, GAD67, is 71% identical in its amino acid sequence but is rarely an autoantigen in T1D [1], [10], [11], interacts differently with the *pyridoxal-5′-phosphate* (PLP) co-factor, and has different kinetics for GABA synthesis in enzyme activity assays [12].

Recently, the crystal structures of human GAD65 and GAD67 were determined [13], and provided a unique insight into the structural basis for autoantigenicity of these closely related isoforms [13], [14], [15]. Analysis of the structures of the protein isoforms has allowed the identification of independent B-cell epitope clusters that locate on opposing faces of the C-terminal domains on GAD65 but not on GAD67 [14]. Structural comparisons revealed two key differences between the isoforms. First, GAD65 is more flexible than GAD67, mainly at the C-terminal domains and at the catalytic loop residues. Second, there are striking differences between these isoforms in their electrostatic charge distribution [15], [16]. These structural and physicochemical differences correlate with known epitope regions in the antigenic isoform GAD65, revealing how the immunodominant epitopes on GAD65 are highly mobile and charged, relative to the corresponding regions in the non-antigenic isoform GAD67 [11], [15], [16]. Although anti-GAD67 antibodies are rare, these antibodies may represent a cross-reactive population of anti-GAD65 [17], [18], but this has not been formally tested. We wondered whether this cross-reactivity might reveal insights into the structural similarities between the isoforms. We therefore set out to more closely examine the reactivity of anti-GAD65 and anti-GAD67 in sera selected to contain anti-GAD65.

## Methods

### Ethics statement

Human sera were originally obtained with written consent, and were derived from previous clinical and epidemiological studies on antibodies to GAD65 approved by the Monash University Human Research Ethics Committee (MUHREC). The sera had been stored without identifying information as a source of control sera to validate new anti-GAD assays, and their use for the present study was approved by MUHREC.

### Sera

Eighty five stored sera that contained anti-GAD65 were selected for study. Selection was based on the availability of sufficient serum for repeat assays and the known presence of anti-GAD65 in the serum. There was a bias towards sera containing high levels of anti-GAD65, considered more likely to contain anti-GAD67, but levels of anti-GAD65 ranged from 30 to >10,000 World Health Organization (WHO) units [19], [20]. Clinical details were limited, but the patients were adults, with T1D or Latent Autoimmune Diabetes of Adults, (LADA) of varying duration. The mouse monoclonal antibody GAD6 [21], [22] was used as a positive control. Ten sera known not to contain anti-GAD65 (2–11 WHO units) from healthy laboratory personnel were also tested, and pooled normal serum from five blood donors was included as a negative control in each assay.

### Radioimmunoprecipitation assay (RIP)

Recombinant human GAD65 and GAD67 (rGAD65, rGAD67) expressed in *Saccharomyces cerevisiae* [23], [24] were used for all experiments. Both proteins were N-terminally truncated, and contained a hexahistidine tag at the C-terminal end to facilitate purification. However both constructs retained full enzyme activity [13], and the GAD65 construct retained all of the reactivity with diabetes sera of full length rGAD65 [23], [24]. Each antigen was iodinated using chloramine T, and anti-GAD levels were measured using a standard radioimmunoprecipitation assay (RIP) previously used in antibody standardization workshops [19], [20]. In brief, 10 µl of serum and 50 µl of ^125^I GAD containing 50,000 cpm were incubated overnight at 4°C. Antigen-antibody complexes were precipitated by the addition of 50 µl of a 50% slurry of Protein A Sepharose (4B conjugate, ZYMED, South San Francisco CA, USA), pelleted by centrifugation, and the percentage of radioactivity in the pellet was calculated.

The specificity of binding of anti-GAD was measured for selected sera by competitive binding experiments in which a 100-fold excess of unlabeled GAD65 or GAD67 (8×10^−9^ M) was added to the standard RIP containing ^125^I labeled GAD65 or GAD67. Homologous cross inhibition was performed using ^125^I GAD65 with unlabeled GAD65 and ^125^I GAD67 with unlabeled GAD67. Heterologous cross inhibition was performed using ^125^I GAD65 with unlabeled GAD67 and ^125^I GAD67 with unlabeled GAD65. All assays were performed in duplicate.

### Anti-GAD affinity measurements

The affinity of anti-GAD67 and/or anti-GAD65 in selected sera was measured by competitive binding experiments based on those described by Mayr et al [25]. For each antigen, the serum was titrated using 10-fold dilutions in the RIP to select a serum dilution at the top of the linear range of the titration curve for use in the affinity assay. This serum dilution was then tested by RIP in which serum was incubated with ^125^I GAD65 or ^125^I GAD67 in the presence of five 10-fold dilutions of unlabeled GAD65 (1.6×10^−11^ M–1.6×10^−7^ M) or GAD67 (1.7×10^−11^ M–1.7×10^−7^ M) or assay buffer only. Following incubation, the assay was completed as for the standard RIP. IC_50_ and *K*_d_ values were calculated by nonlinear regression analysis using GraphPad Prism5 software (GraphPad Software, San Diego, California). Displacement curves were plotted using counts per minutes for each competition reaction on the Y-axis and the logarithmic concentration of unlabeled GAD on the X-axis. The anti-GAD affinity value was expressed as the reciprocal of *K*_d_ (l/mol).

### Measurement of anti-GAD65 and anti-GAD67 by immunoblotting

Recombinant GAD65 and GAD67 separated by 10% SDS PAGE were transferred by electrophoresis to polyvinylidene difluoride (PVDF) membrane, blocked with 4.5% skimmed milk in Tris buffered saline pH 7.5 containing 0.1% Tween 20 (TBST) for one hour and washed three times with TBST. The membrane was incubated with serum diluted 1∶ 500 or 1∶1000 fold in TBST, for 1 hour at room temperature, washed, and incubated with horseradish peroxidase conjugated goat anti-human IgG (Millipore, Melbourne, Australia) or sheep anti-mouse IgG (Millipore), diluted 500 or 1000 fold in TBST. The mouse mAb GAD6, reactive with GAD65, and a normal human serum were included in each test as positive and negative controls. The protein bands were viewed by Amersham Enhanced Chemiluminescence Blotting Detection Reagent (GE Healthcare, Buckingham-shire, UK) with exposure on a Fujifilm Luminescent Image Analyser for one minute.

## Results

### Antibodies to GAD65 and GAD67 detected by RIP

**Figure 1:**
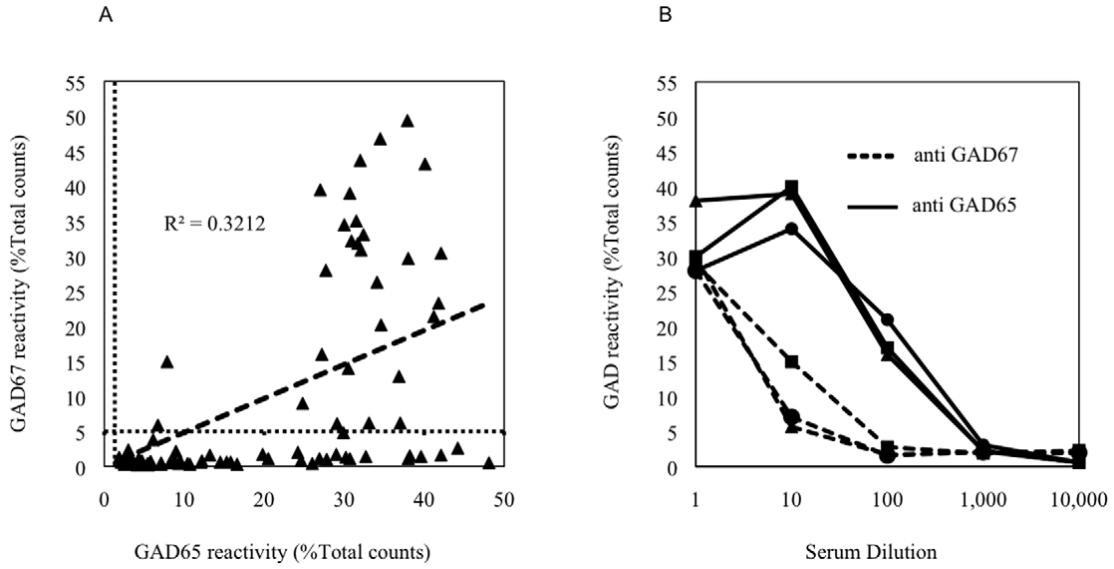
Antibodies to GAD65 and GAD67 detected by RIP. (a) Comparison of antibody reactivity to GAD65 (x-axis) and to GAD67 (y-axis), measured as the percent of the total counts of radioactivity precipitated by each serum (%Total counts) in 85 sera that contain anti-GAD65. The horizontal dotted line indicates the upper limit of normal for anti-GAD67 and the vertical dotted line indicates the upper limit of normal for anti-GAD65, based on mean+3SD of reactivity of 10 normal controls. None of the normal controls showed any reactivity beyond these levels. (b) Titration curves for anti-GAD65 and anti-GAD67 in three representative sera that contain high levels of both anti-GAD65 and anti-GAD67. 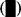, (□) and (▴) represent three different sera samples.

The levels of anti-GAD67 and anti-GAD65 in sera from 85 patients are shown in Figure 1a. For direct comparison the levels of antibodies were expressed as the percent of the total counts of radioactivity (%TC) precipitated by each serum, as the two isoforms contain the same number of tyrosine residues, and the final ^125^I-GAD65 and ^125^I-GAD67 preparations had similar specific activity. The cut-off for positivity for each assay was defined as %TC greater than the mean+3 standard deviations for the 10 healthy controls, which was 1.3 for anti-GAD65, and 5 for anti-GAD67. Using these cut-offs, all 85 sera from patients contained anti-GAD65, and 28 (33%) contained anti-GAD67. For anti-GAD65, there was a close linear relationship between %TC precipitated and WHO units up to ∼20–25%TC (data not shown), which represented approximately 100 WHO units of anti-GAD65, but beyond this the amount of reactivity precipitated began to plateau, and titration was required for quantitative analysis. For many sera that contained the highest levels of anti-GAD65, the %TC precipitated by serum diluted 1∶10 or 1∶100 was equal to or greater than the %TC precipitated by undiluted serum (Figure 1b).

Although there was little correlation between the levels of anti-GAD65 and anti-GAD67 in sera (Figure 1a, R^2^ = 0.321), anti-GAD67 were more common in sera that contained high levels of anti-GAD65, being detected in 25 of 43 (58%) sera with levels of anti-GAD65 >20%TC, but in only 2 of 42 (5%) sera that contained anti-GAD65 <20%TC (P<0.0001). Also, titration indicated that for many sera, the amount of anti-GAD65 was 10–100-fold higher than anti-GAD67 in the same serum (Figure 1b).

### Antibodies to GAD65 and GAD67 by immunoblotting

None of the sera tested for reactivity with GAD65 and GAD67 by immunoblotting reacted strongly with either antigen. Of 23 sera tested, 10 were weakly positive on at least one occasion with GAD65, and 13 were weakly positive with GAD67, but the results showed poor reproducibility. In contrast, the mouse mAb GAD6 that was used as a positive control reacted strongly and consistently with GAD65.

### Measurement of antibody affinity

The affinity of anti-GAD65 and anti-GAD67 in five sera that contained both anti-GAD65 and anti-GAD67 was compared with the affinity of anti-GAD65 in five sera that contained only anti-GAD65 (Table 1). All sera contained high affinity antibodies, ranging from 9×10^9^–9.4×10^10^ l/moles for anti-GAD65, and the dual-reactive sera had an affinity range of 4.8×10^9^–1.5×10^10^ l/moles for anti-GAD67. Although levels of anti-GAD67 were much lower than levels of anti-GAD65 in the sera tested, there were no large differences in the affinity of the two populations, and the affinity was not related to the level of the antibodies present, as judged by the serum dilution at which the affinity was measured (Table 1).

**Table 1:** Affinity of anti-GAD65 and anti-GAD67 in five sera that contain both anti-GAD65 and anti-GAD67 and in five that contain anti-GAD65 only.

Serum	Anti-GAD65			Anti-GAD67		
	Antibody(%TC)	Dilution tested	Affinity(l/mol)	Antibody(%TC)	Dilution tested	Affinity(l/mol)
1	29.8	1 ∶ 500	1×10^10^	42.6	1 ∶ 100	1×10^10^
2	16.6	1 ∶ 100	9×10^9^	11.9	1 ∶ 10	8×10^9^
3	27.8	1 ∶ 100	1×10^10^	21.6	undiluted	4.8×10^10^
4	14.5	1 ∶ 5	6.7×10^10^	15.5	undiluted	1.5×10^10^
5	20.5	1 ∶ 100	9.4×10^10^	27.8	undiluted	1.3×10^10^
6	18.1	1 ∶ 10	1.4×10^10^	2.6	- -	- -
7	12.8	undiluted	2.7×10^10^	0.6	- -	- -
8	7.2	undiluted	1.2×10^10^	0.6	- -	- -
9	5.6	1 ∶ 5	2.2×10^10^	1.6	- -	- -
10	5.5	1 ∶ 10	12.1×10^10^	1.1	- -	- -

### Specificity of antibodies to GAD67

**Figure 2:**
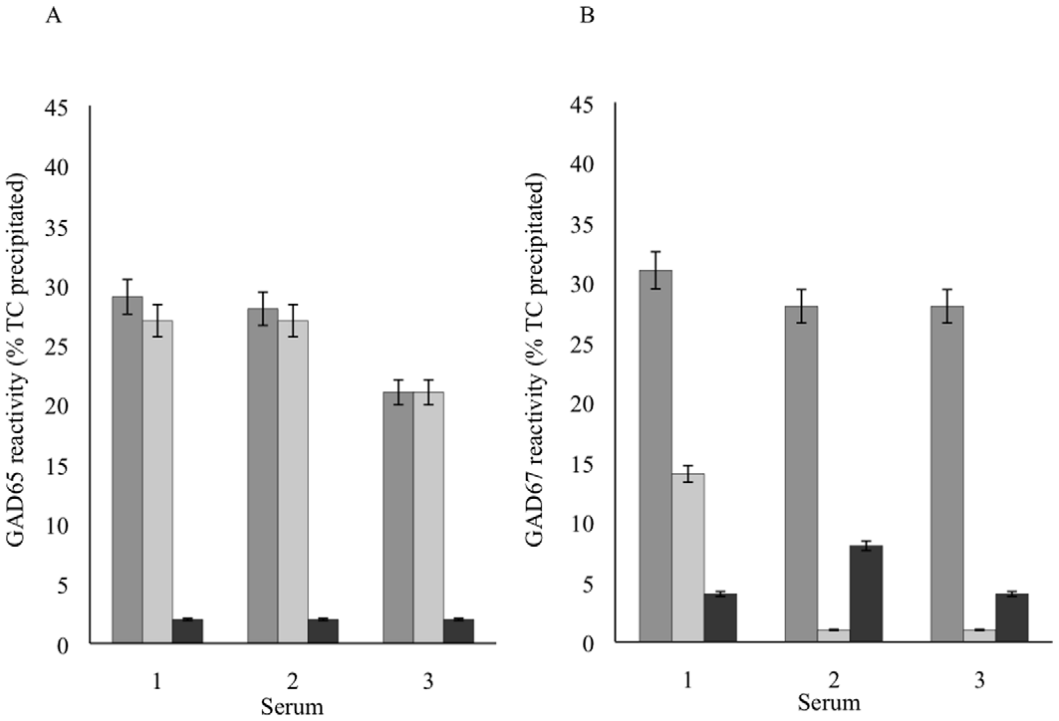
Competition assays to measure cross-reactivity of anti-GAD65 (a) and anti-GAD67 (b). Results, shown as mean and range of duplicates, of inhibition assay for three representative sera inhibited with GAD65 and GAD67. Reactivity with ^125^I-GAD65 or ^125^I-GAD67 (dark grey bars) was inhibited with 100-fold excess of the same GAD (homologous inhibition, black bars). In contrast, unlabelled GAD65 substantially or completely inhibited anti-GAD67, although unlabelled GAD67 did not inhibit anti-GAD65 reactivity (heterologous inhibition, light grey bars).

To determine whether the populations of anti-GAD67 and anti-GAD65 were cross-reactive, 30 sera were tested in heterologous competition assays in which the binding of the radiolabeled GAD65 or GAD67 was inhibited with unlabeled heterologous GAD, and 17 sera were tested both by homologous and heterologous inhibition. Results for three representative sera are shown in Figure 2a,b, and mean results for all sera tested are shown in Table 2. By homologous inhibition, anti-GAD65 was inhibited by unlabeled GAD65, and anti-GAD67 was inhibited by unlabeled GAD67, as expected (Figure 2a,b). In contrast, by heterologous inhibition, anti-GAD67 in most sera was strongly and significantly (P<0.0001) inhibited by unlabeled GAD65, whereas anti-GAD65 was not inhibited by unlabeled GAD67; in almost all cases, inhibition was complete (<2%TC residual activity) except in sera that contained very high levels of antibody (>30%TC) to GAD67. Taken together these results suggested that anti-GAD67 reactivity usually represents a small population of the total anti-GAD65 that react with an epitope shared between the two isoforms. However, there were two exceptional sera. In the first serum that contained both anti-GAD65 (35%TC) and anti-GAD67 (20%TC), anti-GAD67 was completely inhibited by unlabeled GAD65, but unlabeled GAD67 also substantially inhibited anti-GAD65, reducing the reactivity to 9%TC, suggesting that anti-GAD reactive with an epitope present on both GAD65 and GAD67 was a major component of the autoantibody population. For the second serum (anti-GAD65 9%TC; anti-GAD67 17%TC), GAD65 did not inhibit anti-GAD67, although competition with each of the homologous antigens caused complete inhibition, indicating that the two antibody populations were not cross-reactive.

**Table 2:** Results of heterologous inhibition of anti-GAD67 with GAD65, or anti-GAD65 with GAD67 for 30 sera, and of homologous inhibition (italics) of anti-GAD65 with GAD65 and anti-GAD67 with GAD67 for 17 sera, shown as Mean TC% ± SD for each group.

	Antibody	
	Anti-GAD65	Anti-GAD67
**Inhibitor**		
**None**	26.0±12.7*	20.5±13.7**
**GAD65**	*5.0*±*6.8*	4.3±7.5**
**GAD67**	25.0±13.2*	*2.4*±*4.8*

*not significant, dependent t-test.

**p<0.0001, dependent t-test.

## Discussion

In this study we have shown that anti-GAD67 occur in a minority of patients with T1D or LADA who have anti-GAD65. The autoantibodies were readily detected by RIP, but not by immunoblotting indicating that, like anti-GAD65, they reacted with conformational epitopes. Overall anti-GAD67 were rarely detected in sera that did not contain high levels of anti-GAD65, and levels of anti-GAD67 were generally 1–10% of the level of anti-GAD65 in the same serum, but the affinity of binding was similarly high for each population of antibodies. As expected, anti-GAD67 reactivity was readily inhibited by the addition of unlabelled GAD67, but unexpectedly, it was also strongly inhibited by the addition of unlabelled GAD65, whereas anti-GAD65 reactivity was inhibited only with GAD65. Taken together, these results indicate that for most sera anti-GAD67 represents a minor population of anti-GAD65 reactive with an epitope shared between GAD65 and GAD67.

The location of such a cross-reactive epitope is unknown, but several lines of evidence suggest that it is not in the C-terminal domain(s) of GAD65 that have previously been identified as the likely location for major epitopes for anti-GAD65 in diabetes [14]. First, as part of previous epitope mapping studies [26] a mutant was identified in which the amino acids _572_DF_573_ in the C-terminal domain were replaced by alanines. This mutation was thought to cause a major conformational change, particularly in the C-terminus which destroyed the reactivity to GAD65 of the human mAbs b78 and b96.11, and removed anti-GAD65 reactivity from most diabetes sera tested. Interestingly, the diabetes sera in which anti-GAD65 reactivity was not completely removed were sera that contained both anti-GAD65 and anti-GAD67 [26], (and unpublished observations). Further evidence for the lack of a conformational epitope in the C-terminal domain of GAD67 has come from structural studies of a GAD67 mutant that contains the GAD65-catalytic loop (amino acids 423–433). This mutation results in structural changes in the C-terminal domain, but no alteration in anti-GAD67 reactivity (G. Fenalti, personal communication).

The clinical significance of anti-GAD67 in T1D remains unclear. In the present study, sera were selected to contain high levels of anti-GAD65, and according to the availability of sufficient serum, and were not suitable for an unbiased clinical evaluation. However the study does raise the question of the clinical significance of these antibodies. It is important to note that sera from patients with some neurological diseases, particularly stiff person syndrome (SPS), contain high levels of both anti-GAD65 and anti-GAD67 [6], [7], and the anti-GAD reactivity in these patients may differ from that in T1D. Sera from patients with SPS but not T1D generally react strongly by immunoblotting and inhibit GAD enzyme activity [27], [28], and interestingly, anti-GAD reactivity by immunoblotting in SPS-sera is with GAD65 but not GAD67 [28]. Anti-GAD, including both anti-GAD65 and anti-GAD67 can be detected by immunofluorescence on brain in SPS but not in T1D [29], and this anti-GAD67 detected by immunofluorescence is not cross-reactive with GAD65 [30]. Thus, whilst the autoantibodies to GAD65 in SPS do recognise the epitopes that have been defined for sera in T1D, there also appears to be SPS-specific reactivity to other epitopes as well. In several studies using SPS sera a major linear epitope has been identified in the extreme N-terminus of the regulatory sequence in the N-terminal domain of GAD65 that is not detectable in T1D [27], [28], [30], [31], [32], [33], [34]. The results in this present study are based on the experiments conducted on GAD N-terminally truncated by 83-aminoacids, the region of greatest amino acid diversity between the two isoforms [1]. Although the truncated GAD isoforms retain enzyme activity [13] and retain antibody reactivity not only with the sera from TID patients [23], [24], but also with SPS sera [unpublished data], a major linear epitope in SPS has been described within the first 22 amino acids of the N-terminus of GAD65 [27], [35], a region lacking in the GAD65 used in this study.

Our recent study of the physicochemical properties of GAD65 and GAD67 showed that the C-terminal domain of GAD65 that contains the major GAD65 epitopes has much greater flexibility and electrostatic charge than the corresponding region of GAD67, which may explain its greater antigenicity [15]. The same study allowed characterisation and localisation of particular residues within the PLP domains, representing a minor region of flexibility and charge that could represent an epitope region in the PLP-domain that was present in both GAD65 and GAD67. This could be the location of a minor cross-reactive epitope that could be induced by epitope spreading, noting that high affinity antibodies to GAD65 such as those in the present study, have been associated with reactivity with multiple epitopes [25]. Although we have previously identified several physiochemical features of GAD65 such as electrostatic charge, solvation energy and flexibility, that distinguish it from GAD67 as an antigen [15], it is important to recognise that none of these characteristics in isolation are expected to dictate antigenicity. Indeed, the molecular surface of GAD67 shows, although to a much lesser extent than GAD65, some of these characteristics [15], and this may explain the cross reactivity between GAD65 and GAD67.
